# Snail 3G: genomics, genetics, and gene-editing of *Biomphalaria glabrata*

**DOI:** 10.3389/fgene.2026.1721789

**Published:** 2026-01-21

**Authors:** Si-Ming Zhang

**Affiliations:** Center for Evolutionary and Theoretical Immunology, Department of Biology, University of New Mexico, Albuquerque, NM, United States

**Keywords:** *Biomphalaria glabrata*, CRISPR/Cas9, gene-editing, genetic mapping, recombinant inbred line, advanced intercross line, *Schistosoma mansoni*, schistosomiasis

## Abstract

This review highlights recent advances and ongoing challenges in the genomics, genetics, and gene-editing (3G) of the freshwater snail *Biomphalaria glabrata*, based on insights gained from a novel model system we initiated two decades ago. *B. glabrata* is an intermediate host of the human blood fluke *Schistosoma mansoni* and serves as the principal model organism for schistosomiasis research. We developed two homozygous lines of *B. glabrata*, the iM line and iBS90, through 81 and 41 generations of selfing the commonly used M line and BS90, respectively. These lines display contrasting infection phenotypes: susceptibility or resistance to *S. mansoni*. High-quality scaffold-based genome assemblies were generated for both lines, followed by a chromosome-level assembly of the iM line genome. An F_2_ segregating population derived from these lines enabled the identification of three loci, two linked to resistance or susceptibility and one associated with pigmentation, using a double digest restriction-site associated DNA sequencing (ddRADseq) approach. Recombinant inbred lines (RILs) were developed through two crosses and ten generations of selfing. Genetic mapping with RILs refined the resistance locus on chromosome 5 from 8 to 3 Mb through individual-based whole-genome sequencing. Ongoing work includes comparative transcriptome analyses of the two homozygous lines and RILs in response to schistosome infection, along with fine-scale mapping of advanced intercross lines to elucidate the molecular basis of the snail’s anti-schistosome defenses. Over the past 10 years, we have made extensive efforts to achieve germline delivery and generate genetically modified snails. Although pantropic lentiviral and yolk protein–mediated germline delivery methods were unsuccessful, these pioneering experiments provide valuable insights for future research. Finally, we successfully generated germline-edited *B. glabrata*, the first genetically modified schistosomiasis vector snail, by microinjecting CRISPR/Cas9 and guide RNA (gRNA) targeting the *fibrinogen-related protein 3.1* (*FREP3.1*) gene into decapsulated embryos, followed by ex ovo culture. This breakthrough establishes a foundation for innovative genetic strategies to control this neglected tropical disease.

## 1 Introduction

The freshwater snail *Biomphalaria glabrata* (Gastropoda: Planorbidae) is a Neotropical species with 2n = 36 chromosomes and no sex chromosomes (Goldman et al., 1984). This species has been a model organism for schistosomiasis research since the 1950s (Newton, 1953). *B. glabrata* serves as an intermediate host for the human blood fluke *Schistosoma mansoni*, the causative agent of schistosomiasis (bilharziasis), a disease that affects approximately 250 million people in 78 countries (McManus et al., 2018; Lo et al., 2022; Buonfrate et al., 2025). Recently, the disease has been reported in Europe due to globalization and climate change (Gabrielli and Garba Djirmay, 2023; De Vito et al., 2025). Currently, there is no vaccine available against schistosome parasites, and treatment relies solely on praziquantel (PZQ), a chemotherapy that has been in use for about four decades (Seubert et al., 1977). However, reliance on PZQ alone is unlikely to achieve effective disease control or elimination, as reinfection, particularly in children, occurs rapidly after treatment (Wiegand et al., 2017; Kittur et al., 2019; Assaré et al., 2020). Additionally, schistosomes may develop resistance to PZQ, especially during mass drug administration, further complicating its effectiveness in controlling schistosomiasis (Eastham et al., 2024).

Snail control, either alone or combined with other measures, has proven to be one of the most effective strategies for reducing schistosomiasis transmission in endemic areas (King and Bertsch, 2015; Lo et al., 2018). However, the most widely used molluscicide, niclosamide, is toxic to non-target aquatic organisms, limiting its environmental safety and large-scale application (Andrews et al., 1982; King et al., 2015; Zhang et al., 2015; Buddenborg et al., 2019). Since snails play important ecological roles, ideal biocontrol strategies should aim to block the parasite’s intramolluscan development without eradicating snail populations. Supporting this concept, field studies in Brazil have shown that introducing schistosome-resistant *Biomphalaria tenagophila* into endemic regions can reduce transmission (Coelho et al., 2008; Marques et al., 2014).

Genetics-based biocontrol approaches targeting snails, such as CRISPR (clustered regularly interspaced short palindromic repeats)-mediated gene drives, offer a promising alternative for interrupting schistosomiasis transmission (Maier et al., 2019; Grewelle et al., 2022; Pacesa et al., 2024). Similar strategies have shown great promise in controlling mosquito-borne diseases; for instance, genetically modified *Aedes aegypti* mosquitoes have been released in the United States and Brazil to combat arboviral diseases such as dengue, yellow fever, chikungunya, and Zika (Harris et al., 2012; Carvalho et al., 2015; Ernst et al., 2015; Evans et al., 2019; Shaw and Catteruccia, 2019). As gene-edited disease vectors become increasingly important in public health, applying similar strategies to vector snails could transform schistosomiasis control.

Achieving this great goal requires two essential foundations: (1) a thorough understanding of the molecular mechanisms underlying snail resistance to schistosomes and (2) an effective tool for germline gene-editing. This review summarizes our efforts in both areas. Without either foundation, producing and implementing genetically modified snails that target specific genes for disease control is impossible, rendering debates on their field applications premature.

Research on the molecular mechanisms of schistosome resistance in *B. glabrata* has primarily focused on immune responses to infection across various snail strains, generating valuable insights (see recent reviews by Pila et al. (2017), Castillo et al. (2020), Al-Khalaifah (2022), Abou-El-Naga and Mogahed (2024), and Zayed (2025). Unlike those reviews, the present work highlights recent advances and challenges in *B. glabrata* genomics, genetics, and gene-editing, built on unique model systems we began developing two decades ago. The genetic resources developed in this system are timely aligning with cost-effective, high-throughput next-generation sequencing technologies such as Illumina and Pacific Biosciences (PacBio) sequencing, which offer an unprecedented opportunity to explore the biology of vector snails, especially the long-standing question of schistosome resistance.

**FIGURE 1:**
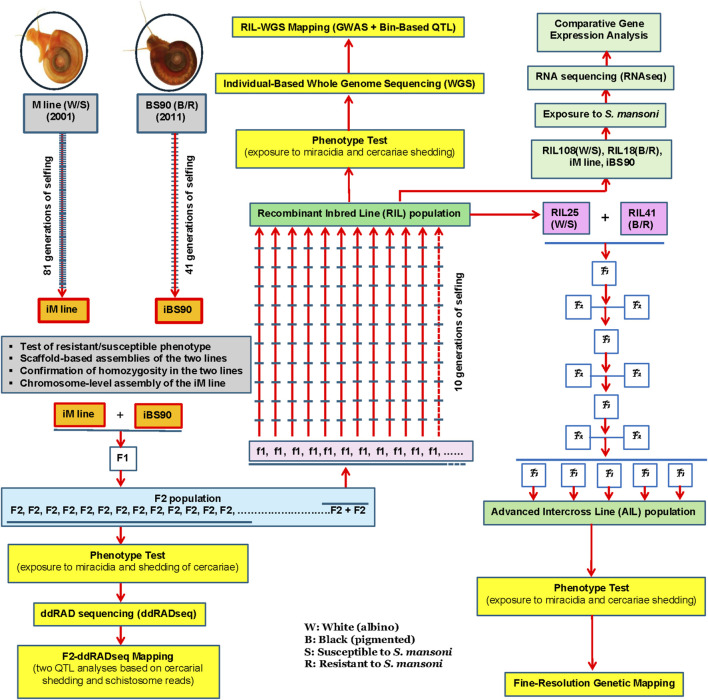
A comprehensive genetic breeding program for *Biomphalaria glabrata* started in 2001.

**FIGURE 2:**
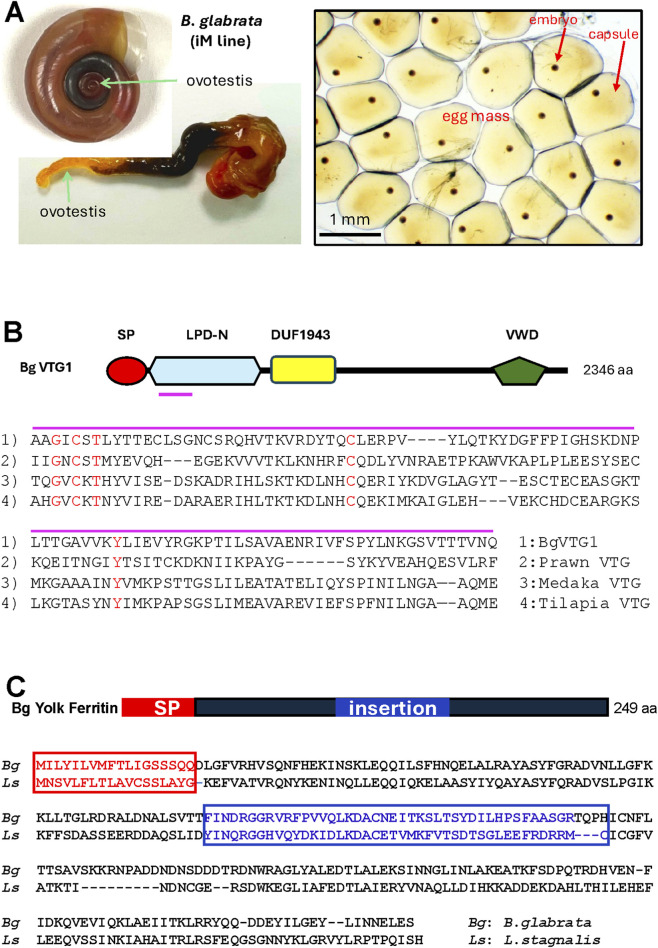
**(A)** Reproductive system of *Biomphalaria glabrata*. **(B)** Structure of BgVtg1 and its alignment with ligand binding to oocyte receptors from various species. A small red line under the BgVtg1 structure indicates the ligand, as shown in the alignment. SP, signal peptide; LPD-N, lipoprotein N-terminal domain; VWD, Von Willebrand factor D. **(C)** Structure of *Biomphalaria glabrata* yolk ferritin and the alignment of yolk ferritins between the snail *Biomphalaria glabrata* (*Bg*) and *Lymnaea stagnalis* (*Ls*).

The first part of this paper, illustrated in Figure 1, provides an overview of the system, including the development of two homozygous lines, whole genome sequencing at the scaffold and chromosome levels, multiple genetic mapping approaches, and comparative transcriptome profiling of different genetic lines in response to schistosome infection. The second part focuses on our efforts in germline delivery and gene-editing, while the pantropic lentiviral and yolk protein–mediated strategies are outlined in Figure 2.

## 2 Development of two homozygous lines of *Biomphalaria glabrata*

Inspired by the significant contributions of animal homozygous lines to life science research, we developed two homozygous *B. glabrata* lines, the iM line and iBS90, derived from the M line and BS90, respectively. The albino M line originated from crosses in the 1950s between albino Brazilian and pigmented Puerto Rican snails (Newton, 1953). The pigmented BS90 snail, also known as the Salvador strain, was isolated in the 1960s from a field population in Salvador, Brazil, and exhibited resistance to both local and allopatric *S. mansoni* (Paraense and Correa, 1963). The M line is susceptible to *S. mansoni*, whereas BS90 is generally resistant; both are commonly used in laboratory research (e.g., Knight et al., 1999; Lewis et al., 2001; Ittiprasert et al., 2010; Li et al., 2020; Lu et al., 2022; Blouin et al., 2024).

The development of the iM line began in 2001, followed by the iBS90 in 2011. The iM line was generated through 81 generations of selfing from a single M line snail, while iBS90 was produced by 41 generations of selfing from a single BS90 snail (Figure 1; Bu et al., 2022). The homozygous breeding programs for the iM line and iBS90 were completed in 2018 and 2022, respectively, and the populations have since been maintained at the Center for Evolutionary and Theoretical Immunology (CETI) at the University of New Mexico (UNM) (Bu et al., 2022; Zhang et al., 2024; Zhong et al., 2024).

We first tested the infection phenotype of the two lines based on cercarial shedding; snails shedding cercariae were considered susceptible, while those that did not were regarded as resistant to schistosome infection (see Section 4.1 for more details). Our repeated tests showed that the iM line consistently exhibited susceptibility to the New World South American PR1 and NMRI strains of *S. mansoni* acquired from the NIH/NIAID-funded Schistosomiasis Resource Center of the Biomedical Research Institute (www.afbr-bri.org), while iBS90 was completely resistant. Similar results were obtained when both lines were challenged with field-derived Old World Kenyan *S. mansoni*, suggesting that genetic loci conferring broad-spectrum resistance are likely conserved across geographically distinct snail populations.

Next, we assessed the homozygosity of the two lines using K-mer analysis of their Illumina reads and confirmed both lines are homozygous (Bu et al., 2022). These characteristics make the iM line and iBS90 powerful models for genomic and genetic studies, enabling highly controlled, accurate, and reproducible experiments.

## 3 Whole genome sequencing of *Biomphalaria glabrata*

### 3.1 Scaffold-based genome assembly

To empower the two homozygous lines for genetic mapping and other biological research, we first sequenced their complete mitochondrial genomes (Zhang et al., 2018), followed by their nuclear genomes (Bu et al., 2022). The mitochondrial genomes are 13,667 nucleotides (nt) for the iM line and 13,676 nt for iBS90, with a sequence identity of 94.5% (Zhang et al., 2018).

For the nuclear genomes, two individuals from each line were sequenced using Illumina and PacBio platforms, respectively (Bu et al., 2022). The assembled haploid genome sizes were 871 million bases (Mb) for the iM line (120× coverage; 255 scaffolds; N50 = 22.7 Mb) and 885 Mb for iBS90 (87× coverage; 346 scaffolds; N50 = 19.4 Mb). Benchmarking Universal Single-Copy Orthologs (BUSCO) analysis indicated 96% genome completeness for both assemblies. These results represent a substantial improvement over the first *B. glabrata* reference genome from the BBO2 strain, which contained more than 331,400 scaffolds, had 88.8% BUSCO completeness, and an N50 of 48 kb (Adema et al., 2017).

Our comparative whole-genome analysis revealed extensive single nucleotide polymorphisms (SNPs) and small structural variants, but no evidence of large-scale subchromosomal rearrangements between the iM line and iBS90 genomes (Bu et al., 2022). This suggests that *S. mansoni* resistance or susceptibility is unlikely to be driven by major chromosomal rearrangements.

### 3.2 Chromosome-level genome assembly

To further improve assembly quality, we applied Omni-C sequencing for scaffolding and generated the first chromosome-level genome assembly for *B. glabrata* (iM line), achieving a total of 337× coverage after combining all sequencing datasets (Zhong et al., 2024). This yielded 18 sequence contact matrices corresponding to the 18 haploid chromosomes (2n = 36), with 96.5% of scaffold sequences anchored to these chromosomes. The eighteen chromosome assemblies (Zhong et al., 2024) were largely validated by the 18 genetic linkage groups we previously reported (Bu et al., 2022).

The iM line genome is predicted to encode 34,559 protein-coding genes and 2,406 non-coding RNAs, with 42.5% repetitive elements (Zhong et al., 2024). Because the iM line is completely homozygous, all homologous chromosomes are identical, allowing the assembly to represent a single haploid genome rather than a heterozygous composite commonly found in current genome assemblies. This eliminates the haplotype resolution issues associated with heterozygous organisms and improves sequence reliability, highlighting the advantages of using homozygous lines for whole genome sequencing.

The final assembled genome size was 842,576,133 bp, closely matching a later report of 845,861,586 bp (Berriman et al., 2024), a difference of only 0.4%. This size is slightly smaller than our earlier scaffold-based assembly of 871 Mb (Bu et al., 2022) and substantially smaller than the initial BBO2 draft genome of 916 Mb (Adema et al., 2017). Based on these findings, we estimate the true assembled genome size to be 843–846 Mb. Our flow cytometry measurement of the haploid DNA content (C-value) for the iM line is 1.11 pg, which is equivalent to approximately 1,086 Mb in genome size (Bu et al., 2023; Zhang et al., 2025). This indicates that the genome assembly is about 22.4% smaller than the DNA content–based estimate, a common discrepancy due to the challenges of assembling repetitive sequences (Elliott and Gregory, 2015). It is important to note that the current assembly and annotation require refinement, especially considering the over 20 years of significant effort to finalize the human genome sequence (Nurk et al., 2022). More advanced genomic technologies, such as telomere-to-telomere (T2T) genome assembly combined with long-read assembled transcriptomes, could enable the generation of a gapless, chromosome-level assembled genome with high-quality annotation (Nurk et al., 2022; Nip et al., 2023; Li and Durbin, 2024).

To evaluate the assembly’s application, we mapped two major immune-related gene families—toll-like receptor (*TLR*) genes and fibrinogen-related domain–containing protein (*FReD*) genes—onto the 18 assembled chromosomes. A total of 70 *TLR*-like genes were found across 13 chromosomes. The three chromosomes with the highest number of *TLRs* are chromosome 14 (34.3%; 24/70), chromosome 8 (18.6%; 13/70), and chromosome 15 (15.7%; 11/70). The remaining 10 chromosomes each hosted 1 to 5 *TLRs*, collectively accounting for 31.4% of the total. We identified 80 *FReD* genes across 15 chromosomes, with chromosome 13 exhibiting the highest proportion of *FReDs* (44 of 80; 55%). Notably, nearly all fibrinogen-related protein (*FREP*) genes, characterized by the presence of both immunoglobulin (IgSF) and fibrinogen domain sequences, were located in a compact region: 39 of 40 *FREP* genes lie within a ∼5 Mb segment of chromosome 13, which spans ∼37 Mb (Zhong et al., 2024). In addition to these inherent loci, recombination, mutation, and alternative splicing, along with retrotransposition, may play a role in enhancing *FREP* diversity (Zhang and Loker, 2003; Zhang et al., 2004; Hanington and Zhang, 2011). Clustering in a compact genomic region may facilitate genetic exchange among related genes, promoting *FREP* sequence diversification.

## 4 Genetic mapping and comparative transcriptome profiling

### 4.1 F_2_-ddRADseq mapping

For genetic mapping studies, we first generated an F_2_ segregating population using the homozygous iM line and iBS90 snails as parents, followed by selfing the F_1_ snails (Figure 1; Bu et al., 2022). A total of 1,133 juvenile F_2_ snails from this population were individually exposed to *S. mansoni*. Finally, we successfully phenotyped 869 individuals for resistance/susceptibility and pigmentation/albinism. Among these, 421 were resistant and 448 were susceptible. A Chi-square goodness-of-fit test indicated that the ratio of resistance to susceptibility significantly deviated from the expected 3:1 Mendelian ratio, suggesting that schistosome resistance in juvenile snails is not controlled by a single gene (χ^2^ = 18.78, P < 0.01). This result aligns with earlier findings from classical genetic crosses (Richards, 1970; Richards et al., 1992; Lewis et al., 2001).

We utilized F_2_ samples with recorded body color for genetic studies, as no prior genetic mapping of pigmentation in snails has been reported, and pigmentation markers have important implications for basic research. A total of 674 F_2_ snails were pigmented, and 195 were albino (χ^2^ = 3.04, P = 0.08), consistent with a previous observation suggesting that pigmentation is controlled by a single locus (Richards, 1975). There was no association between pigmentation and resistance or susceptibility.

A subset of 126 F_2_ individuals was genotyped using double digest restriction-site associated DNA sequencing (ddRADseq), a reduced-representation approach that typically samples 1%–2% of the genome (Peterson et al., 2012). Snail DNA was digested with *EcoR* I and *Msp* I, followed by paired-end Illumina sequencing, a strategy we refer to as the F_2_-ddRADseq approach (Bu et al., 2022).

In all genetic mapping studies, we focused on juvenile snails (3–5 mm in shell diameter) because they are more susceptible than adults and play a significant role in transmission (Richards, 1970). The phenotype of resistance or susceptibility to schistosomes in snails was determined by infection outcomes, specifically cercarial shedding, rather than infection status or miracidia penetration (Bu et al., 2022; Zhang et al., 2024). Cercariae are the human-infective stage and are directly responsible for disease transmission and human infection; therefore, we focused on cercarial shedding as the phenotype, aligning it with field relevance and disease control. *Biomphalaria glabrata* typically begins shedding cercariae approximately 36 days post-infection and continues for months. In our studies, susceptible snails were those actively shedding cercariae, while resistant snails were identified 2 months after exposure if no shedding occurred.

This approach identified two quantitative trait loci (QTLs) associated with resistance or susceptibility: qRS-2.1 on chromosome 2 and qRS-5.1 on chromosome 5. The resistance QTL qRS-5.1 exhibited an additive effect of 0.30 (increased resistance when inherited from the resistant parent), while the susceptibility QTL qRS-2.1 had a dominant effect of 0.41 (reduced resistance when inherited from the susceptible parent).

We also used the abundance of schistosome reads from DNA extracted from whole snails as an alternative phenotype. Reads that did not map to the snail genome but aligned to the *S. mansoni* genome provided an estimate of parasite load. Higher parasite read counts indicated greater snail susceptibility. QTLs (qsm-2.1 and qsm-5.1) derived from this metric corresponded closely to those identified using cercarial shedding, confirming the robustness of our mapping results.

The two resistance/susceptibility QTLs span 0.5–1.43 cM genetically (0.99–1.36 Mb physically), but their 95% confidence intervals are larger (2–8 cM; 4.03–21.36 Mb). Both loci are situated in centromeric or pericentromeric regions, characterized by low recombination rates, which may influence resistance or susceptibility in *B. glabrata*.

Finally, using the same dataset with two phenotypes—infection and pigmentation—we performed the first genetic mapping of snail pigmentation. One major QTL (qBC-13.1) was identified on chromosome 13, with a high logarithm of the odds (LOD) score (8.92) and an additive effect of 0.34 (Bu et al., 2022).

### 4.2 RIL-WGS mapping

As a healthy *B. glabrata* can produce thousands of eggs/embryos over half a year, we established a recombinant inbred line (RIL) population using the remaining F_2_ snails from our earlier F_2_-ddRADseq mapping experiment (Figure 1; Zhang et al., 2024). RILs arise from multiple meiotic crossovers, creating a mosaic of parental genomes, and subsequent selfing increases recombination events, thereby reducing heterozygosity (Broman, 2005; Pollard, 2012). Therefore, RIL populations provide higher mapping resolution than F_2_ populations, which have limited recombination and large linkage blocks.

Our RIL snails were generated through a parental outcross followed by an F_2_ intercross and ten generations of self-fertilization, yielding more recombination events than standard RIL schemes (Takuno et al., 2012) (Figure 1). From 338 starting F_2_ pairs, we ultimately obtained 118 recombinant inbred lines (RILs) with distinct infection phenotypes.

We applied individual-based whole-genome sequencing (WGS) at 16× coverage to 46 phenotyped lines, half resistant (n = 23) and half susceptible, to scan the genome. As expected, this RIL-WGS approach reduced the resistance QTL interval from ∼8 Mb in the F_2_ snails to ∼3 Mb in the RILs. Furthermore, it revealed additional minor-effect loci requiring further validation. Genome-wide association study (GWAS) and bin-marker QTL mapping consistently identified a 3 Mb resistance locus, BgSRR1, on chromosome 5 (Zhang et al., 2024), in agreement with our F_2_-ddRADseq results (Bu et al., 2022). These data indicate that chromosome 5 harbors major resistance determinants in *B. glabrata*.

Within BgSRR1, we identified 118 protein-coding genes. Many have unknown functions, but some are associated with known cellular or humoral immunity. For example, one gene encodes mitogen-activated protein kinase (MAPK), which is involved in hemocyte-mediated encapsulation and H_2_O_2_ production that kills intramolluscan schistosomes (Humphries et al., 2001; Zelck et al., 2007). We also identified ficolin-like genes characterized solely by fibrinogen domain sequences (Bidula et al., 2019), which have not previously been implicated in *B. glabrata* defense. Additionally, *immune response gene 1* (encoding cis-aconitate decarboxylase) was detected, suggesting a role in immunometabolism (Michelucci et al., 2013). The involvement of immunometabolism has also been noted in the *Bulinus* spp.–*Schistosoma haematobium* system (Habib et al., 2024a; Lekired et al., 2025). Functional analysis of genes within BgSRR1 may reveal novel anti-schistosome defense mechanisms that have not been previously recognized.

### 4.3 Development of advanced intercross lines (AILs)

To further increase mapping resolution, we have recently generated advanced intercross lines (AILs) using RIL snails as described above. This is our final effort to create genetic resources for this >20-year genetic breeding program. The AILs were derived by crossing two RIL snails with contrasting phenotypes. After eight additional generations of crossing and selfing, these AILs accumulated substantially more recombination events, providing a powerful resource for fine-scale genetic mapping (Figure 1). The genetic mapping of the AIL snails is in progress.

### 4.4 Summary of genetic mapping strategy

In laboratory-based biological models, genetic mapping of a trait is, in principle, an association study between phenotype and genotype. The reliability of these results largely depends on well-defined phenotypes, high-resolution genotyping, and large sample sizes. If a study involves ambiguous phenotypes, less sensitive genotyping, or small sample sizes (or limited genome coverage), the findings may be questionable, regardless of the analytical methods applied. With this in mind, we utilized large sample sizes with distinct phenotypes related to disease transmission and carefully selected high-resolution genotyping approaches for complementary investigation.

Critically, we employed well-designed genetic mapping populations rather than existing laboratory strains. The mapping populations—F_2_, RIL, and AIL—are derived from two homozygous parental lines (iM line and iBS90), whose genome sequences are completed and publicly available. The chromosome-level assembly of the iM line serves as the reference genome. These populations are pedigree-connected, have known origins, retain parental genetic elements, and display contrasting phenotypes (resistance vs. susceptibility; albino vs. pigmented). All genotype and phenotype datasets have been deposited in public repositories, ensuring that the mapping results are fully traceable, reproducible, and accessible for reanalysis.

### 4.5 Comparative transcriptome profiling

To leverage genetic resources and understand global gene expression in different genetic lines with different genotypes and phenotypes in response to schistosome infection, we have conducted comparative transcriptome analyses using two complementary models (Figure 1). The two models encompass four genetic lines with contrasting infection phenotypes in response to *S. mansoni* infection. The first model includes two homozygous lines: the susceptible iM line and the resistant iBS90 line. The second model comprises two RILs, one resistant and the other susceptible to *S. mansoni*, mirroring the two homozygous lines. Each model represents a distinct genetic background but displays opposite infection phenotypes (resistance vs. susceptibility). By systematically analyzing gene expression patterns across these models, we aim to identify genes and pathways consistently associated with resistance, as well as potential differences in defense strategies employed by genetically distinct lines. Data analysis is currently underway.

## 5 Germline delivery and germline gene-editing

### 5.1 Daunting challenges in generating genetically modified live snails

Producing live genetically modified animals requires germline modification—targeting sperm, eggs, or embryos—so that the resulting embryos carrying the mutation can develop into viable juveniles. Germline modification technologies in animals have evolved from classical transgenesis, first reported in the early 1980s (Gordon et al., 1980; Gordon and Ruddle, 1981), to cutting-edge CRISPR gene-editing technology used today (Hwang et al., 2013; Ernst et al., 2015; Evans et al., 2019; D’Amato et al., 2024). Remarkably, despite decades of effort, no schistosomiasis vector snail had been successfully germline-modified until our recent report describing the first gene-edited *B. glabrata* snail (Oonuma et al., 2025; see Section 5.4).

This persistent challenge stemmed from the unique reproductive biology of planorbid snails (family Planorbidae), particularly the genera *Biomphalaria* and *Bulinus*, which together are responsible for more than 95% of global schistosomiasis transmission (Gordon et al., 2019). These snails are hermaphroditic but preferentially outcross. Both male and female gametes develop within a single organ, the ovotestis (Figure 2A). Fertilization occurs internally with either allosperm or autosperm, rendering *in vitro* fertilization and direct manipulation of gametes, such as via electroporation, impractical (Jarne et al., 2010).

Microinjection, a standard method in early transgenic animal research and many current germline editing studies, is technically feasible but extremely challenging in planorbid snails. It must be performed on fragile, minute, decapsulated embryos (∼0.1 mm in diameter) immediately after egg laying. Removing the capsule compromises both mechanical protection and albumen fluid, which are vital for nutrition and antimicrobial defense. Consequently, decapsulated embryos are highly vulnerable to mechanical damage and microbial infection, leading to very low survival rates in ex ovo culture and posing major obstacles to generating germline-edited live snails.

In brief, the primary reason for the unsuccessful generation of genetically modified snails is the lack of effective technology for delivering genetic materials (e.g., transgenes) into germline cells, known as germline delivery, due to the unique characteristics of the snail reproductive system. Genetic editing materials, such as CRISPR cargo (Cas9 and gRNA), can function in all cell types across different organisms and modify germline genomes as intended. Once the challenge of germline delivery is overcome, the potential to produce various types of genetically modified snails will emerge, similar to what has been achieved in other animals.

### 5.2 Lentivirus-mediated germline delivery

Our initial strategy for developing germline delivery was to test whether pantropic lentiviruses could effectively deliver transgenes into snail gametes. Lentiviruses are non-replicating, self-inactivating pseudoviruses derived from human immunodeficiency virus (HIV) and pseudotyped with vesicular stomatitis virus glycoprotein (VSV-G). The versatile VSV-G has a broad tropism, enabling to infect various cell types, including male and female gametes across a wide phylogenetic spectrum (Brindley and Pearce, 2007; Core et al., 2012; Chen et al., 2016; Naldini et al., 2016). Once the lentivirus enters the target cell, the transgene located between two LTRs derived from HIV integrates into the host genome, with the LTRs flanking the transgene facilitating stable genomic integration.

We constructed two recombinant lentiviruses carrying the green fluorescent protein (GFP) gene under the control of either the *Actin 1* or *Actin 2* gene promoter. Promoters were amplified from *B. glabrata* genomic DNA by PCR and inserted into the pLVX-ZsGreen vector (Creative Biogene. www.creative-biogene.com), replacing the original cytomegalovirus (CMV) promoter. Viral particles were then produced using standard recombinant lentivirus protocols.

High-titer viruses (10^8^–10^9^ TU/mL) were injected into the ovotestis of adult albino iM line snails (5–10 µL per snail) using a WPI manual micromanipulator (www.wpiinc.com). Injected snails were housed individually in 1 L plastic cups and allowed to self-fertilize to produce F_1_ offspring for evaluation.

Successful genomic integration would be indicated by PCR amplification in F_1_ offspring, and functional promoter activity would produce observable GFP fluorescence. Despite extensive optimization of viral concentration, injection volume, and injection technique, we found no reliable evidence of transgene integration after examining more than 400 F_1_ offspring. Occasional false-positive PCR signals observed likely arose from residual viral particles or free viral DNA in the aquatic environment. Furthermore, intrinsic GFP-like autofluorescence in *B. glabrata* complicated detection. These issues underscore the need for rigorous validation to avoid misinterpretation.

### 5.3 Yolk protein-mediated germline delivery

Our next strategy for germline delivery was to explore a natural reproductive mechanism—oocyte receptor-mediated endocytosis—by using snail yolk proteins as potential carriers for transgene delivery into developing oocytes. In oviparous animals like insects and mollusks, oocytes accumulate large amounts of yolk proteins during oogenesis to support embryonic development. Major yolk proteins, such as vitellogenins, are synthesized in non-gonadal tissues, secreted into the hemolymph, and internalized by oocytes through receptor-mediated endocytosis, a highly conserved biological process (Raikhel and Dhadialla, 1992; Snigirevskaya et al., 1997; Biscotti et al., 2018).

Through proteomic, transcriptomic, phylogenetic, and bioinformatic analyses, we characterized yolk proteins in *B. glabrata* (Habib et al., 2024b). Vitellogenin 1 (BgVtg1) was identified as the most abundant yolk protein in the ovotestis, while yolk ferritin was less abundant (Habib et al., 2024b). This finding revised earlier assumptions that yolk ferritin predominates in *Biomphalaria*, as it is the major yolk protein in *Planorbarius corneus* and *Lymnaea stagnalis*, both of which are phylogenetically closely related to *Biomphalaria* (Bottke and Sinha, 1979; Bottke et al., 1988). Despite its lower abundance, yolk ferritin remained a candidate carrier for transgene delivery.

During receptor-mediated endocytosis, only a small subset of yolk proteins (ligands) binds to specific oocyte membrane receptors to trigger yolk protein uptake. We identified putative receptor-binding domains in BgVtg1 by aligning its amino acid sequence with known oocyte-binding ligands from the vitellogenins of the prawn *Macrobrachium rosenbergii*, medaka *Oryzias latipes*, and blue tilapia *Oreochromis aureus* (Li et al., 2003; Roth et al., 2013; Murakami et al., 2019) (Figure 2B). Biochemical and computational analyses suggest that these ligands facilitate receptor binding and activate endocytosis.

Yolk ferritin differs structurally from cytoplasmic ferritins by possessing a unique 42–amino acid insertion within the B–C loop of the ferritin subunit that is exposed on the protein surface (Bottke et al., 1988; Andrews et al., 1992) (Figure 2C). This insertion was hypothesized to act analogously to vitellogenin’s oocyte-binding region, functioning as a ligand for receptor-mediated uptake and thereby representing a promising target for recombinant protein design.

We cloned nucleotide sequences encoding the two ligand domains from BgVtg1 and yolk ferritin into a bacterial expression vector (pET Biotin His6-mCherry LIC) to generate recombinant fusion proteins containing the ligand and an mCherry fluorescent tag. The recombinant proteins were highly purified, and a free mCherry control was included. These proteins were injected into the ovotestis of adult iM line snails at varying concentrations and volumes. Due to the open circulatory system, the injected proteins enter the hemolymph and reach developing oocytes. Successful receptor-mediated uptake would be indicated by increased mCherry fluorescence within oocytes compared with surrounding tissues and control snails injected with free mCherry.

Fluorescence microscopy of dissected ovotestes under different experimental setups revealed no detectable increase in mCherry signal in oocytes relative to surrounding tissues or controls, indicating a lack of uptake. Notably, a similar strategy—Receptor-Mediated Ovary Transduction of Cargo (ReMOT Control)—has been successfully developed in arthropods (Chaverra-Rodriguez et al., 2018; Xu et al., 2020; Terradas et al., 2023). Our unsuccessful attempts with both lentiviral and yolk protein-mediated germline delivery suggest the need for more rigorous experimentation and highlight the complex physical, biochemical, and possibly immunological barriers in *B. glabrata* oocytes and ovotestis, which remain poorly understood.

### 5.4 Generation of the first gene-edited *Biomphalaria glabrata* using CRISPR/Cas9

Finally, we successfully delivered genetic materials into the germline cells of *B. glabrata* and generated germline-modified live snails using CRISPR/Cas9 gene-editing technology, a powerful tool for targeted gene disruption and gene drive development (Hwang et al., 2013; Carvalho et al., 2015; Evans et al., 2019; Pacesa et al., 2024). This was achieved by microinjecting Cas9 mRNA and guide RNA (gRNA) targeting the *fibrinogen-related protein 3.1* (*FREP3.1*) gene into decapsulated embryos, followed by ex ovo culture (Oonuma et al., 2025). Although CRISPR-mediated gene editing in *B. glabrata* has been reported for somatic gene editing in embryonic cell lines (Bge) (Coelho et al., 2020) and epigenetic modification in embryos (Luviano et al., 2022), our study represents the first successful application of CRISPR technology for germline gene-editing in schistosomiasis vector snails, marking the creation of the first genetically modified and inheritable live snail in this context (Oonuma et al., 2025).

We used pre-iBS90 snails (the 31st generation of selfed BS90) (Bu et al., 2022) because of their stable resistance to *S. mansoni* and nearly homozygous genetic background. *FREP3.1* knockout was confirmed by heteroduplex mobility assay (HMA) and Sanger sequencing. Stable germline transmission was achieved, and two homozygous *FREP3.1* knockout lines were established (Oonuma et al., 2025).

Functional assays revealed that loss of *FREP3.1* did not abolish resistance to *S. mansoni*, contradicting our initial hypothesis about its anti-schistosome role (Oonuma et al., 2025). While *FREP3.2* has been shown to contribute to defense against trematode infection (Zhang et al., 2001; Hanington et al., 2010; Mitta et al., 2017; Li et al., 2020), *FREP3.1* belongs to a distinct subfamily based on nucleotide sequence identity (79% identity), despite their similar names (Zhang and Loker, 2003; Oonuma et al., 2025). This distinction, together with our results, underscores the complexity of the *FREP* gene family and suggests potential functional redundancy. Compensatory upregulation of other *FREPs* or immune factors may obscure the phenotype (Barbaric et al., 2007; Peng, 2019; Salanga and Salanga, 2021). Alternatively, *FREP3.1* may have a non-essential or context-dependent role, consistent with our earlier hypothesis regarding the diverse functional repertoire of the *FREP* gene family (Zhang et al., 2008a, 2008b; Hanington and Zhang, 2011).

This gene-editing study marks a milestone in snail functional genomics. The function of genes in snails has primarily been evaluated using RNA interference (RNAi) (Jiang et al., 2006; Hanington et al., 2010; Knight et al., 2011). RNAi-mediated knockdown temporarily suppresses the expression of target genes, often resulting in subtle or ambiguous phenotypes. CRISPR-mediated knockout or knock-in creates heritable, complete gene disruptions or introduces new gene functions through genome insertions, offering a definitive approach to functional analysis. More importantly, this breakthrough lays the groundwork for developing gene drive systems in schistosome-transmitting snails, a potentially transformative strategy for controlling snail-borne schistosomiasis (Oonuma et al., 2025).

## 6 Concluding remarks

Our investigation of snail 3G—genomics, genetics, and gene-editing in *B. glabrata*—is a long-term, step-by-step process that involves painstaking efforts. While our approach is time-consuming and labor-intensive, it enables rigorous data validation, effective tracking, and iterative refinement. By establishing two homozygous lines, generating scaffold- and chromosome-level genome assemblies, creating and analyzing multi-generational genetic mapping populations, and conducting comparative transcriptomic studies, we have built a strong platform for dissecting the genetic architecture underlying schistosome resistance and other key biological traits.

Our genetic mapping studies consistently implicate resistance loci on chromosome 5, although discrepancies remain compared to results from other laboratories using different strains and mapping strategies (Knight et al., 1999; Tennessen et al., 2015, 2020; Blouin et al., 2024). These differences likely reflect genetic variation among strains, methodological differences, or the involvement of multiple loci (Richards et al., 1992; Lewis et al., 2001), underscoring the complexity of host–parasite interactions (Bu et al., 2022; Zhang et al., 2024). While our findings are derived from a well-controlled laboratory system, further studies, particularly under field conditions, are needed to validate and extend these observations. Additionally, comprehensive functional characterization of candidate genes remains a high priority.

Our unsuccessful attempts at using lentiviral and yolk protein–mediated germline delivery should not be interpreted as evidence that these methods are unfeasible in *B. glabrata*. Rather, they highlight the need for deeper insights into snail reproductive biology and greater experimental investment. Although microinjection is a highly skilled and technically demanding process, the successful demonstration of CRISPR/Cas9-mediated germline editing in *B. glabrata* through microinjection provides proof of principle for targeted genetic interventions. This advance opens new avenues for interrupting disease transmission while preserving ecological integrity, marking a significant step forward in the fight against schistosomiasis.

Snail 3G, particularly functional genomics, is still in its infancy compared to well-established disease vector systems like mosquitoes. Substantial work lies ahead to achieve the ultimate goal: Snail-targeted genetic control of schistosomiasis in the developing world.

## References

[B1] Abou-El-Naga I. F. Mogahed N. M. F. H. (2024). Immuno-molecular profile for biomphalaria glabrata/schistosoma mansoni interaction. Dev. Comp. Immunol. 150, 105083. 10.1016/j.dci.2023.105083 37852455

[B2] Adema C. M. Hillier L. W. Jones C. S. Loker E. S. Knight M. Minx P. (2017). Whole genome analysis of a schistosomiasis-transmitting freshwater snail. Nat. Commun. 8, 15451. 10.1038/ncomms15451 28508897 PMC5440852

[B3] Al-Khalaifah H. (2022). Cellular and humoral immune response between snail hosts and their parasites. Front. Immunol. 13, 981314. 10.3389/fimmu.2022.981314 36439176 PMC9685329

[B4] Andrews P. Thyssen J. Lorke D. (1982). The biology and toxicology of molluscicides, bayluscide. Pharmacol. Ther. 19 (2), 245–295. 10.1016/0163-7258(82)90064-x 6763710

[B5] Andrews S. C. Arosio P. Bottke W. Briat J. F. von Darl M. Harrison P. M. (1992). Structure, function, and evolution of ferritins. J. Inorg. Biochem. 47 (3-4), 161–174. 10.1016/0162-0134(92)84062-r 1431878

[B6] Assaré R. K. N’Tamon R. N. Bellai L. G. Koffi J. A. Mathieu T. B. I. Ouattara M. (2020). Characteristics of persistent hotspots of *Schistosoma mansoni* in Western Côte d’Ivoire. Parasites Vectors 13, 1. 10.1186/s13071-020-04188-x 32616074 PMC7333430

[B7] Barbaric I. Miller G. Dear T. N. (2007). Appearances can be deceiving: phenotypes of knockout mice. Brief. Funct. Genomics Proteomics 6 (2), 91–103. 10.1093/bfgp/elm008 17584761

[B8] Berriman M. Buddenborg S. and Wellcome Sanger Institute Tree of Life Management, Samples and Laboratory Team (2024). The genome sequence of the bloodfluke planorb, *Biomphalaria glabrata* (Say, 1818). Wellcome Open Res. 9, 435. 10.12688/wellcomeopenres.22819.1 PMC13000397 41869676

[B9] Bidula S. Sexton D. W. Schelenz S. (2019). Ficolins and the recognition of pathogenic microorganisms: an overview of the innate immune response and contribution of single nucleotide polymorphisms. J. Immunol. Res. 2019, 3205072. 10.1155/2019/3205072 30868077 PMC6379837

[B10] Biscotti M. A. Barucca M. Carducci F. Canapa A. (2018). New perspectives on the evolutionary history of vitellogenin gene family in vertebrates. Genome Biol. Evol. 10 (10), 2709–2715. 10.1093/gbe/evy206 30239716 PMC6185446

[B11] Blouin M. S. Bollmann S. R. Le Clec'h W. Chevalier F. D. Anderson T. J. C. Tennessen J. A. (2024). Susceptibility of BS90 *Biomphalaria glabrata* snails to infection by SmLE *Schistosoma mansoni* segregates as a dominant allele in a cluster of polymorphic genes for single-pass transmembrane proteins. PLoS Negl. Trop. Dis. 18 (9), e0012474. 10.1371/journal.pntd.0012474 39283952 PMC11426442

[B12] Bottke W. Sinha I. (1979). Ferritin as an exogenous yolk protein in snails. Wilehm Roux. Arch. Dev. Biol. 186 (1), 71–75. 10.1007/BF00848109 28305313

[B13] Bottke W. Burschyk M. Volmer J. (1988). On the origin of the yolk protein ferritin in snails. Rouxs Arch. Dev. Biol. 197 (7), 377–382. 10.1007/BF00398988 28305744

[B14] Brindley P. J. Pearce E. J. (2007). Genetic manipulation of schistosomes. Int. J. Parasitol. 37 (5), 465–473. 10.1016/j.ijpara.2006.12.012 17280677

[B15] Broman K. W. (2005). The genomes of recombinant inbred lines. Genetics 169 (2), 1133–1146. 10.1534/genetics.104.035212 15545647 PMC1449115

[B16] Bu L. Zhong D. Lu L. Loker E. S. Yan G. Zhang S.-M. (2022). Compatibility between snails and schistosomes: insights from new genetic resources, comparative genomics, and genetic mapping. Commun. Biol. 5 (1), 940. 10.1038/s42003-022-03844-5 36085314 PMC9463173

[B17] Bu L. Lu L. Laidemitt M. R. Zhang S.-M. Mutuku M. Mkoji G. (2023). A genome sequence for *Biomphalaria pfeifferi*, the major vector snail for the human-infecting parasite *Schistosoma mansoni* . PLoS Negl. Trop. Dis. 17 (3), e0011208. 10.1371/journal.pntd.0011208 36961841 PMC10075465

[B18] Buddenborg S. K. Kamel B. Bu L. Zhang S.-M. Mkoji G. M. Loker E. S. (2019). Transcriptional responses of *Biomphalaria pfeifferi* and *Schistosoma mansoni* following exposure to niclosamide, with evidence for a synergistic effect on snails following exposure to both stressors. PLoS Negl. Trop. Dis. 13 (12), e0006927. 10.1371/journal.pntd.0006927 31841501 PMC6936870

[B19] Buonfrate D. Ferrari T. C. A. Adegnika A. A. Russell Stothard J. Gobbi F. G. (2025). Human schistosomiasis. Lancet 405 (10479), 658–670. 10.1016/S0140-6736(24)02814-9 39986748

[B20] Carvalho D. O. McKemey A. R. Garziera L. Lacroix R. Donnelly C. A. Alphey L. (2015). Suppression of a field population of *Aedes aegypti* in Brazil by sustained release of transgenic male mosquitoes. PLoS Negl. Trop. Dis. 9 (7), e0003864. 10.1371/journal.pntd.0003864 26135160 PMC4489809

[B21] Castillo M. G. Humphries J. E. Mourão M. M. Marquez J. Gonzalez A. Montelongo C. E. (2020). *Biomphalaria glabrata* immunity: post-Genome advances. Dev. Comp. Immunol. 104, 103557. 10.1016/j.dci.2019.103557 31759924 PMC8995041

[B22] Chaverra-Rodriguez D. Macias V. M. Hughes G. L. Pujhari S. Suzuki Y. Peterson D. R. (2018). Targeted delivery of CRISPR-Cas9 ribonucleoprotein into arthropod ovaries for heritable germline gene editing. Nat. Commun. 9 (1), 3008. 10.1038/s41467-018-05425-9 30068905 PMC6070532

[B23] Chen X. Y. Zhu Z. W. Yu F. X. Huang J. Hu X. R. Pan J. Z. (2016). Production of germline transgenic pigs co-expressing double fluorescent proteins by lentiviral vector. Anim. Reprod. Sci. 174, 11–19. 10.1016/j.anireprosci.2016.05.009 27639503

[B24] Coelho P. M. Rosa F. M. Maciel E. Negrão-Correa D. A. Carvalho O. S. Caldeira R. L. (2008). Transmission control of schistosomiasis mansoni by introduction of a resistant strain of *Biomphalaria tenagophila* in areas where transmission is maintained by this species. Acta Trop. 108 (2-3), 245–248. 10.1016/j.actatropica.2008.05.028 18598664

[B25] Coelho F. S. Rodpai R. Miller A. Karinshak S. E. Mann V. H. Dos Santos Carvalho O. (2020). Diminished adherence of *Biomphalaria glabrata* embryonic cell line to sporocysts of *Schistosoma mansoni* following programmed knockout of the allograft inflammatory factor. Parasit. Vectors 13 (1), 511. 10.1186/s13071-020-04384-9 33050923 PMC7552541

[B26] Core A. B. Reyna A. E. Conaway E. A. Bradham C. A. (2012). Pantropic retroviruses as a transduction tool for sea urchin embryos. Proc. Natl. Acad. Sci. U. S. A. 109 (14), 5334–5339. 10.1073/pnas.1117846109 22431628 PMC3325703

[B27] De Vito A. Colpani A. Moi G. Moné H. Mouahid G. Fusco D. (2025). Risk of autochthonous human schistosomiasis transmission in Italy. Infez. Med. 33 (3), 279–283. 10.53854/liim-3303-4 40933223 PMC12419172

[B28] D’Amato R. Taxiarchi C. Galardini M. Trusso A. Minuz R. L. Grilli S. (2024). Anti-CRISPR *Anopheles* mosquitoes inhibit gene drive spread under challenging behavioral conditions in large cages. Nat. Commun. 15, 952. 10.1038/s41467-024-44907-x 38296981 PMC10830555

[B29] Eastham G. Fausnacht D. Becker M. H. Gillen A. Moore W. (2024). Praziquantel resistance in schistosomes: a brief report. Front. Parasitol. 3, 1471451. 10.3389/fpara.2024.1471451 39817170 PMC11732111

[B30] Elliott T. A. Gregory T. R. (2015). What’s in a genome? The C-value enigma and the evolution of eukaryotic genome content. Philoso. Trans. R. Soc. Lond. Ser. B Biol. Sci. 370, 20140331. 10.1098/rstb.2014.0331 26323762 PMC4571570

[B31] Ernst K. C. Haenchen S. Dickinson K. Doyle M. S. Walker K. Monaghan A. J. (2015). Awareness and support of release of genetically modified “sterile” mosquitoes, key West, Florida, USA. Emerg. Infect. Dis. 21 (2), 320–324. 10.3201/eid2102.141035 25625795 PMC4313646

[B32] Evans B. R. Kotsakiozi P. Costa-da-Silva A. L. Sayuri Ioshino E. S. Garxiera L. Pedrosa M. C. (2019). Transgenic *Aedes aegypti* mosquitoes transfer genes into a natural population. Sci. Rep. 9, 13047. 10.1038/s41598-019-49660-6 31506595 PMC6736937

[B33] Gabrielli A. F. Garba Djirmay A. (2023). Schistosomiasis in Europe. Curr. Trop. Med. Rep. 10, 79–87. 10.1007/s40475-023-00286-9

[B34] Goldman M. A. LoVerde P. T. Chrisman C. L. Frankin D. A. (1984). Chromosomal evolution in planorbid snails of the genus *Bulinus* and *Biomphalaria* . Malcologia 25 (2), 427–446.

[B35] Gordon J. W. Ruddle F. H. (1981). Integration and stable germ line transmission of genes injected into mouse pronuclei. Science 214 (4526), 1244–1246. 10.1126/science.6272397 6272397

[B36] Gordon J. W. Scangos G. A. Plotkin D. J. Barbosa J. A. Ruddle F. H. (1980). Genetic transformation of mouse embryos by microinjection of purified DNA. Proc. Natl. Acad. Sci. U. S. A. 77 (12), 7380–7384. 10.1073/pnas.77.12.7380 6261253 PMC350507

[B37] Gordon C. A. Kurscheid J. Williams G. M. Clements A. C. A. Li Y. Zhou X. N. (2019). Asian schistosomiasis: current status and prospects for control leading to elimination. Trop. Med. Infect. Dis. 4 (1), 40. 10.3390/tropicalmed4010040 30813615 PMC6473711

[B38] Grewelle R. E. Perez-Saez J. Tycko J. Namigai E. K. O. Rickards C. G. De Leo G. A. (2022). Modeling the efficacy of CRISPR gene drive for snail immunity on schistosomiasis control. PLoS Negl. Trop. Dis. 16 (10), e0010894. 10.1371/journal.pntd.0010894 36315503 PMC9648845

[B39] Habib M. R. Posavi M. Lekired A. Zhang S.-M. (2024a). Exploring the genome-wide transcriptomic responses of *Bulinus truncatus* to *Schistosoma haematobium* infection: an important host-parasite system involved in the transmission of human urogenital schistosomiasis. Mol. Immunol. 175, 74–88. 10.1016/j.molimm.2024.09.006 39307031 PMC12019995

[B40] Habib M. R. Bu L. Posavi M. Zhong D. Yan G. Zhang S.-M. (2024b). Yolk proteins of the schistosomiasis vector snail *Biomphalaria glabrata* revealed by multi-omics analysis. Sci. Rep. 14 (1), 1820. 10.1038/s41598-024-52392-x 38245605 PMC10799875

[B41] Hanington P. C. Zhang S.-M. (2011). The primary role of fibrinogen-related proteins in invertebrates is defense, not coagulation. J. Innate. Immun. 3 (1), 17–27. 10.1159/000321882 21063081 PMC3031514

[B42] Hanington P. C. Forys M. A. Dragoo J. W. Zhang S.-M. Adema C. M. Loker E. S. (2010). Role for a somatically diversified lectin in resistance of an invertebrate to parasite infection. Proc. Natl. Acad. Sci. U. S. A. 107 (49), 21087–21092. 10.1073/pnas.1011242107 21084634 PMC3000291

[B43] Harris A. F. McKemey A. R. Nimmo D. Curtis Z. Black I. Morgan S. A. (2012). Successful suppression of a field mosquito population by sustained release of engineered male mosquitoes. Nat. Biotechnol. 30 (9), 828–830. 10.1038/nbt.2350 22965050

[B44] Humphries J. E. Elizondo L. Yoshino T. P. (2001). Protein kinase C regulation of cell spreading in the molluscan *Biomphalaria glabrata* embryonic (Bge) cell line. Biochim. Biophys. Acta. 1540 (3), 243–252. 10.1016/s0167-4889(01)00136-7 11583819

[B45] Hwang W. Y. Fu Y. Reyon D. Maeder M. L. Tsai S. Q. Sander J. D. (2013). Efficient genome editing in zebrafish using a CRISPR-Cas system. Nat. Biotechnol. 31 (3), 227–229. 10.1038/nbt.2501 23360964 PMC3686313

[B46] Ittiprasert W. Miller A. Myers J. Nene V. El-Sayed N. M. Knight M. (2010). Identification of immediate response genes dominantly expressed in juvenile resistant and susceptible *Biomphalaria glabrata* snails upon exposure to *Schistosoma mansoni* . Mol. Biochem. Parasitol. 169 (1), 27–39. 10.1016/j.molbiopara.2009.09.009 19815034 PMC2785114

[B47] Jarne P. David P. Pointier J.-P. Koene J. M. (2010). “Basommatophoran gastropods,” in The evolution of primary sexual characters in animals (New York: Oxford University Press), 173–196.

[B48] Jiang Y. Loker E. S. Zhang S.-M. (2006). *In vivo* and *in vitro* knockdown of *FREP2* gene expression in the snail *Biomphalaria glabrata* using RNA interference. Dev. Comp. Immunol. 30 (10), 855–866. 10.1016/j.dci.2005.12.004 16442620 PMC3641767

[B49] King C. H. Bertsch D. (2015). Historical perspective: snail control to prevent schistosomiasis. PLoS Negl. Trop. Dis. 9 (4), e0003657. 10.1371/journal.pntd.0003657 25905621 PMC4408102

[B50] King C. H. Sutherland L. J. Bertsch D. (2015). Systematic review and meta-analysis of the impact of chemical-based mollusciciding for control of *Schistosoma mansoni* and *S. haematobium* transmission. PLoS Negl. Trop. Dis. 9 (12), e0004290. 10.1371/journal.pntd.0004290 26709922 PMC4692485

[B51] Kittur N. King C. Campbell C. Kinung’hi S. Mwinzi P. Karanja D. (2019). Persistent hotspots in schistosomiasis consortium for operational research and evaluation studies for gaining and sustaining control of schistosomiasis after four years of mass drug administration of praziquantel. Am. J. Trop. Med. Hyg. 3, 617–627. 10.4269/ajtmh.19-0193 31287046 PMC6726953

[B52] Knight M. Miller A. N. Patterson C. N. Rowe C. G. Michaels G. Carr D. (1999). The identification of markers segregating with resistance to *Schistosoma mansoni* infection in the snail *Biomphalaria glabrata* . Proc. Natl. Acad. Sci. U. S. A. 96 (4), 1510–1515. 10.1073/pnas.96.4.1510 9990054 PMC15498

[B53] Knight M. Miller A. Liu Y. Scaria P. Woodle M. Ittiprasert W. (2011). Polyethyleneimine (PEI) mediated siRNA gene silencing in the *Schistosoma mansoni* snail host, *Biomphalaria glabrata* . PLoS Negl. Trop. Dis. 5 (7), e1212. 10.1371/journal.pntd.0001212 21765961 PMC3134429

[B54] Lekired A. Mudge J. Laidemitt M. R. Mutuku M. W. Loker E. S. Zhang S.-M. (2025). Whole-genome sequencing and genome-wide transcriptome profiling of the freshwater planorbid snail *Bulinus ugandae* (Mollusca: gastropoda), a Nilotic bulinine refractory to *Schistosoma haematobium* . BMC Genomics 26, 1089. 10.1186/s12864-025-12320-3 41361373 PMC12690972

[B55] Lewis F. A. Patterson C. N. Knight M. Richards C. S. (2001). The relationship between *Schistosoma mansoni* and *Biomphalaria glabrata:* genetic and molecular approaches. Parasitology 123 (Suppl. l), S169–S179. 10.1017/s0031182001007831 11769281

[B56] Li H. Durbin R. (2024). Genome assembly in the telomere-to-telomere era. Nat. Rev. Genet. 25 (9), 658–670. 10.1038/s41576-024-00718-w 38649458

[B57] Li A. Sadasivam M. Ding J. L. (2003). Receptor-ligand interaction between vitellogenin receptor (VtgR) and vitellogenin (Vtg), implications on low density lipoprotein receptor and apolipoprotein B/E. The first three ligand-binding repeats of VtgR interact with the amino-terminal region of Vtg. J. Biol. Chem. 278 (5), 2799–2806. 10.1074/jbc.M205067200 12429745

[B58] Li H. Hambrook J. R. Pila E. A. Gharamah A. A. Fang J. Wu X. (2020). Coordination of humoral immune factors dictates compatibility between *Schistosoma mansoni* and *Biomphalaria glabrata* . Elife 9, e51708. 10.7554/eLife.51708 31916937 PMC6970513

[B59] Lo N. C. Gurarie D. Yoon N. Coulibaly J. T. Bendavid E. Andrews J. R. (2018). Impact and cost-effectiveness of snail control to achieve disease control targets for schistosomiasis. Proc. Natl. Acad. Sci. U. S. A. 115 (4), E584–E591. 10.1073/pnas.1708729114 29301964 PMC5789907

[B60] Lo N. C. Bezerra F. S. M. Colley D. G. Fleming F. M. Homeida M. Kabatereine N. (2022). Review of 2022 WHO guidelines on the control and elimination of schistosomiasis. Lancet Infect. Dis. 22 (11), e327–e335. 10.1016/S1473-3099(22)00221-3 35594896

[B61] Lu L. Bu L. Zhang S. -M. Buddenborg S. K. Loker E. S. (2022). An overview of transcriptional responses of schistosome-susceptible (M line) or -resistant (BS-90) *Biomphalaria glabrata* exposed or not to *Schistosoma mansoni* infection. Front. Immunol. 12, 805882. 10.3389/fimmu.2021.805882 35095891 PMC8791074

[B62] Luviano N. Duval D. Ittiprasert W. Allienne J. F. Tavernier G. Chaparro C. (2022). Hit-and-run epigenetic editing for vectors of snail-borne parasitic diseases. Front. Cell. Dev. Biol. 10, 794650. 10.3389/fcell.2022.794650 35295851 PMC8920497

[B63] Maier T. Wheeler N. J. Namigai E. K. O. Tycko J. Grewelle R. E. Woldeamanuel Y. (2019). Gene drives for schistosomiasis transmission control. PLoS Negl. Trop. Dis. 13 (12), e0007833. 10.1371/journal.pntd.0007833 31856157 PMC6922350

[B64] Marques D. P. Rosa F. M. Maciel E. Negrão-Corrêa D. Teles H. M. Caldeira R. L. (2014). Reduced susceptibility of a *Biomphalaria tenagophila* population to *Schistosoma mansoni* after introducing the resistant Taim/RS strain of *B. tenagophila* into herivelton martins stream. PLoS One 9 (6), e99573. 10.1371/journal.pone.0099573 24941324 PMC4062407

[B65] McManus D. P. Dunne D. W. Sacko M. Utzinger J. Vennervalid B. J. Zhou X.-N. (2018). Schistosomiasis. Nat. Rev. Dis. Prim. 4, 13. 10.1038/s41572-018-0013-8 30093684

[B66] Michelucci A. Cordes T. Ghelfi J. Pailot A. Reiling N. Goldmann O. (2013). Immune-responsive gene 1 protein links metabolism to immunity by catalyzing itaconic acid production. Proc. Natl. Acad. Sci. U. S. A. 110 (19), 7820–7825. 10.1073/pnas.1218599110 23610393 PMC3651434

[B67] Mitta G. Gourbal B. Grunau C. Knight M. Bridger J. M. Théron A. (2017). The compatibility between *Biomphalaria glabrata* snails and *Schistosoma mansoni*: an increasingly complex puzzle. Adv. Parasitol. 97, 111–145. 10.1016/bs.apar.2016.08.006 28325369

[B68] Murakami Y. Horibe T. Kinoshita M. (2019). Development of an efficient bioreactor system for delivering foreign proteins secreted from liver into eggs with a vitellogenin signal in medaka *Oryzias latipes* . Fish. Sci. 85, 677–685. 10.1007/s12562-019-01320-4

[B69] Naldini L. Trono D. Verma I. M. (2016). Lentiviral vectors, two decades later. Science 353 (6304), 1101–1102. 10.1126/science.aah6192 27609877

[B70] Newton W. L. (1953). The inheritance of susceptibility to infection with *Schistosoma mansoni* in *Australorbis glabratus* . Exp. Parasitol. 2, 242–257. 10.1016/0014-4894(53)90036-8

[B71] Nip K. M. Hafezqorani S. Gagalova K. K. Chiu R. Yang C. Warren R. L. (2023). Reference-free assembly of long-read transcriptome sequencing data with RNA-Bloom2. Nat. Commun. 14, 2940. 10.1038/s41467-023-38553-y 37217540 PMC10202958

[B72] Nurk S. Koren S. Rhie A. Rautiainen M. Bzikadze A. V. Mikheenko A. (2022). The compete sequence of a human genome. Science 376 (6588), 44–53. 10.1126/science.abj6987 35357919 PMC9186530

[B73] Oonuma K. Kuroda R. Uchida T. Zhang S.-M. (2025). CRISPR/Cas9-germline editing of *Biomphalaria glabrata*: a breakthrough in genetic modification of snails that transmit schistosomiasis. Sci. Adv. 11 (41), eadx5889. 10.1126/sciadv.adx5889 41061057 PMC12506961

[B74] Pacesa M. Pelea O. Jinek M. (2024). Past, present, and future of CRISPR genome editing technologies. Cell 187 (5), 1076–1100. 10.1016/j.cell.2024.01.042 38428389

[B75] Paraense W. L. Correa L. R. (1963). Variation in susceptibility of populations of *Australorbis glabratus* to a strain of *Schistosoma mansoni* . Rev. Inst. Med. Trop. Sao Paulo. 5, 15–22. 13941355

[B76] Peng J. (2019). Gene redundancy and gene compensation: an updated view. J. Genet. Genomics 46 (7), 329–333. 10.1016/j.jgg.2019.07.001 31377237

[B77] Peterson B. K. Weber J. N. Kay E. H. Fisher H. S. Hoekstra H. E. (2012). Double digest RADseq: an inexpensive method for *de novo* SNP discovery and genotyping in model and non-model species. PLoS One 7 (5), e37135. 10.1371/journal.pone.0037135 22675423 PMC3365034

[B78] Pila E. A. Li H. Hambrook J. R. Wu X. Hanington P. C. (2017). Schistosomiasis from a snail's perspective: advances in snail immunity. Trends Parasitol. 33 (11), 845–857. 10.1016/j.pt.2017.07.006 28803793

[B79] Pollard D. A. (2012). Design and construction of recombinant inbred lines. Methods Mol. Biol. 871, 31–39. 10.1007/978-1-61779-785-9_3 22565831

[B80] Raikhel A. S. Dhadialla T. S. (1992). Accumulation of yolk proteins in insect oocytes. Annu. Rev. Entomol. 37, 217–251. 10.1146/annurev.en.37.010192.001245 1311540

[B81] Richards C. S. (1970). Genetics of a molluscan vector of schistosomiasis. Nature 227, 806–810. 10.1038/227806a0 5432242

[B82] Richards C. S. (1975). Genetics of pigmentation in *Biomphalaria straminea* . Am. J. Trop. Med. Hyg. 24, 154–156. 10.4269/ajtmh.1975.24.154 1111353

[B83] Richards C. S. Knight M. Lewis F. A. (1992). Genetics of *Biomphalaria glabrata* and its effect on the outcome of *Schistosoma mansoni* infection. Parasitol. Today 8 (5), 171–174. 10.1016/0169-4758(92)90015-t 15463608

[B84] Roth Z. Weil S. Aflalo E. D. Manor R. Sagi A. Khalaila I. (2013). Identification of receptor-interacting regions of vitellogenin within evolutionarily conserved β-sheet structures by using a peptide array. Chembiochem 14 (9), 1116–1122. 10.1002/cbic.201300152 23733483

[B85] Salanga C. M. Salanga M. C. (2021). Genotype to phenotype: CRISPR gene editing reveals genetic compensation as a mechanism for phenotypic disjunction of morphants and mutants. Int. J. Mol. Sci. 22 (7), 3472. 10.3390/ijms22073472 33801686 PMC8036752

[B86] Seubert J. Pohlke R. Loebich F. (1977). Synthesis and properties of praziquantel, a novel broad spectrum anthelmintic with excellent activity against schistosomes and cestodes. Experientia 33 (8), 1036–1037. 10.1007/BF01945954 891804

[B87] Shaw W. R. Catteruccia F. (2019). Vector biology meets disease control: using basic research to fight vector-borne diseases. Nat. Microbiol. 4 (1), 20–34. 10.1038/s41564-018-0214-7 30150735 PMC6437764

[B88] Snigirevskaya E. S. Hays A. R. Raikhel A. S. (1997). Secretory and internalization pathways of mosquito yolk protein precursors. Cell Tissue Res. 290 (1), 129–142. 10.1007/s004410050915 9377633

[B89] Takuno S. Terauchi R. Innan H. (2012). The power of QTL mapping with RILs. PLoS One 7 (10), e46545. 10.1371/journal.pone.0046545 23056339 PMC3467243

[B90] Tennessen J. A. Bonner K. M. Bollmann S. R. Johnstun J. A. Yeh J. Y. Marine M. (2015). Genome-wide scan and test of candidate genes in the snail *Biomphalaria glabrata* reveal new locus influencing resistance to *Schistosoma mansoni* . PLoS Negl. Trop. Dis. 9 (9), e0004077. 10.1371/journal.pntd.0004077 26372103 PMC4570800

[B91] Tennessen J. A. Bollmann S. R. Peremyslova E. Kronmiller B. A. Sergi C. Hamali B. (2020). Clusters of polymorphic transmembrane genes control resistance to schistosomes in snail vectors. Elife 9, e59395. 10.7554/eLife.59395 32845238 PMC7494358

[B92] Terradas G. Macias V. M. Peterson H. McKeand S. Krawczyk G. Rasgon J. L. (2023). The development and expansion of *in vivo* germline editing technologies in arthropods: receptor-mediated ovary transduction of cargo (ReMOT control) and beyond. Integr. Comp. Biol. 63 (6), 1550–1563. 10.1093/icb/icad123 37742320 PMC10755176

[B93] Wiegand R. E. Mwinzi P. N. M. Montgomery S. P. Chan Y. L. Andiego K. Omedo M. (2017). A persistent hotspot of *Schistosoma mansoni* infection in a five-year randomized trial of praziquantel preventative chemotherapy strategies. J. Infect. Dis. 11, 1425–1433. 10.1093/infdis/jix496 28968877 PMC5913648

[B94] Xu S. Pham T. Neupane S. (2020). Delivery methods for CRISPR/Cas9 gene editing in crustaceans. Mar. Life Sci. Technol. 2 (1), 1–5. 10.1007/s42995-019-00011-4 33313574 PMC7731668

[B95] Zayed K. M. (2025). Innate and putative adaptive immunological responses of schistosome-parasitized snails. Acta Trop. 261, 107503. 10.1016/j.actatropica.2024.107503 39675412

[B96] Zelck U. E. Gege B. E. Schmid S. (2007). Specific inhibitors of mitogen-activated protein kinase and PI3-K pathways impair immune responses by hemocytes of trematode intermediate host snails. Dev. Comp. Immunol. 31 (4), 321–331. 10.1016/j.dci.2006.06.006 16926049

[B97] Zhang S.-M. Loker E. S. (2003). The *FREP* gene family in the snail *Biomphalaria glabrata*: additional members, and evidence consistent with alternative splicing and *FREP* retrosequences. Dev. Comp. Immunol. 27 (3), 175–187. 10.1016/s0145-305x(02)00091-5 12590969

[B98] Zhang S.-M. Léonard P. M. Adema C. M. Loker E. S. (2001). Parasite-responsive IgSF members in the snail *Biomphalaria glabrata*: characterization of novel genes with tandemly arranged IgSF domains and a fibrinogen domain. Immunogenetics 53 (8), 684–694. 10.1007/s00251-001-0386-8 11797103

[B99] Zhang S.-M. Adema C. M. Kepler T. B. Loker E. S. (2004). Diversification of Ig superfamily genes in an invertebrate. Science 305 (5681), 251–254. 10.1126/science.1088069 15247481

[B100] Zhang S.-M. Zeng Y. Loker E. S. (2008a). Expression profiling and binding properties of fibrinogen-related proteins (FREPs), plasma proteins from the schistosome snail host *Biomphalaria glabrata* . Innate Immun. 14 (3), 175–189. 10.1177/1753425908093800 18562576 PMC3638879

[B101] Zhang S.-M. Nian H. Zeng Y. Dejong R. J. (2008b). Fibrinogen-bearing protein genes in the snail *Biomphalaria glabrata*: characterization of two novel genes and expression studies during ontogenesis and trematode infection. Dev. Comp. Immunol. 32 (10), 1119–1130. 10.1016/j.dci.2008.03.001 18417215 PMC2585491

[B102] Zhang S.-M. Buddenborg S. K. Adema C. M. Sullivan J. T. Loker E. S. (2015). Altered gene expression in the schistosome-transmitting snail *Biomphalaria glabrata* following exposure to niclosamide, the active ingredient in the widely used molluscicide Bayluscide. PLoS Negl. Trop. 9 (10), e0004131. 10.1371/journal.pntd.0004131 26452273 PMC4599737

[B103] Zhang S.-M. Bu L. Laidemitt M. R. Lu L. Mutuku M. W. Mkoji G. M. (2018). Complete mitochondrial and rDNA complex sequences of important vector species of *Biomphalaria*, obligatory hosts of the human-infecting blood fluke, *Schistosoma mansoni* . Sci. Rep. 8 (1), 7341. 10.1038/s41598-018-25463-z 29743617 PMC5943310

[B104] Zhang S.-M. Yan G. Lekired A. Zhong D. (2024). Genomic basis of schistosome resistance in a molluscan vector of human schistosomiasis. iScience 28 (1), 111520. 10.1016/j.isci.2024.111520 39758819 PMC11699755

[B105] Zhang S.-M. Adema C. M. Habib M. R. Lekired A. Posavi M. Laidemitt M. R. (2025). Complexity of schistosome vector bulinine snails in Kenya: insights from nuclear genome size variation, complete mitochondrial genome sequence, and morphometric analysis. PLoS Negl. Trop. Dis. 19 (7), e0013305. 10.1371/journal.pntd.0013305 40658733 PMC12274006

[B106] Zhong D. Bu L. Habib M. R. Lu L. Yan G. Zhang S.-M. (2024). A haplotype-like, chromosome-level assembled and annotated genome of *Biomphalaria glabrata*, an important intermediate host of schistosomiasis and the best studied model of schistosomiasis vector snails. PLoS Negl. Trop. Dis. 18 (2), e0011983. 10.1371/journal.pntd.0011983 38421953 PMC10903818

